# Forest defoliator pests alter carbon and nitrogen cycles

**DOI:** 10.1098/rsos.160361

**Published:** 2016-10-26

**Authors:** Anne l-M-Arnold, Maren Grüning, Judy Simon, Annett-Barbara Reinhardt, Norbert Lamersdorf, Carsten Thies

**Affiliations:** 1 Institute of Soil Science of Temperate and Boreal Ecosystems, Büsgen-Institute, Georg-August-University, Büsgenweg 2, 37077 Göttingen, Germany; 2 Department of Biology, Plant Physiology and Biochemistry Group, University of Konstanz Universitätsstrasse 10, 78457 Konstanz, Germany; 3 Natural Resources Research Laboratory, Bremer Str. 15, 29308 Winsen, Germany

**Keywords:** forest pest, climate change, disturbance, temperate forest, defoliation, C- and N-cycle

## Abstract

Climate change may foster pest epidemics in forests, and thereby the fluxes of elements that are indicators of ecosystem functioning. We examined compounds of carbon (C) and nitrogen (N) in insect faeces, leaf litter, throughfall and analysed the soils of deciduous oak forests (*Quercus petraea* L.) that were heavily infested by the leaf herbivores winter moth (*Operophtera brumata* L.) and mottled umber (*Erannis defoliaria* L.). In infested forests, total net canopy-to-soil fluxes of C and N deriving from insect faeces, leaf litter and throughfall were 30- and 18-fold higher compared with uninfested oak forests, with 4333 kg C ha^−1^ and 319 kg N ha^−1^, respectively, during a pest outbreak over 3 years. In infested forests, C and N levels in soil solutions were enhanced and C/N ratios in humus layers were reduced indicating an extended canopy-to-soil element pathway compared with the non-infested forests. In a microcosm incubation experiment, soil treatments with insect faeces showed 16-fold higher fluxes of carbon dioxide and 10-fold higher fluxes of dissolved organic carbon compared with soil treatments without added insect faeces (control). Thus, the deposition of high rates of nitrogen and rapidly decomposable carbon compounds in the course of forest pest epidemics appears to stimulate soil microbial activity (i.e. heterotrophic respiration), and therefore, may represent an important mechanism by which climate change can initiate a carbon cycle feedback.

## 1. Introduction

Insect pests in forests have formerly been recognized as discrete biotic disturbance occurring at relatively small spatial scales and short time intervals. However, in the view of climate change, outbreaks arise with unprecedented extent and severity [1–3]. During outbreaks, widespread defoliation is associated with considerable fluxes of organic matter and nutrients via insect faeces, litter fragments and throughfall (precipitation). Such element fluxes in forests are rarely considered in biogeochemical analyses (or included only in a condensed form in large-scale operations) [4–6], but are expected to influence ecosystem functioning by modifying microbial growth and organic matter decomposition, and thereby their feedback to plants and other ecosystem components.

Feedback effects between plants and nutrient cycles represent an important mechanism by which herbivores can mediate changes in ecosystem functioning [7]. The ‘acceleration hypothesis’ predicts that herbivores have an accelerating effect on nutrient cycling, while plant responses include compensatory growth, increased nutrient uptake and plant productivity (e.g. [8]). This subsequently results in increased decomposition and nutrient turnover in the ecosystem, and a positive feedback favouring plants. The ‘deceleration hypothesis’ (e.g. [9]) predicts that herbivores have a decelerating effect on nutrient cycling, with herbivores selectively feeding on nutrient-rich plants, and thereby increasing the abundance of nutrient-poor (and chemically or physically defended) plants. This subsequently results in decreased decomposition and nutrient turnover and a reduced productivity from plants. There are several indications for the functioning of both mechanisms in plant communities (e.g. [8–11]). Yet, the effects of herbivory on nutrient cycling are not sufficiently understood to predict ecosystem effects. The lack of knowledge on the biogeochemical consequences of pest outbreak populations attacking millions of hectares of forested land annually contributes further to this unpredictability [2,4,5,12].

In this 3-year study, we analysed the biogeochemical consequences of outbreak populations of the winter moth (*Operophtera brumata* L.) and the mottled umber (*Erannis defoliaria* L.), both of which are dominant herbivores in deciduous oak forests (*Quercus petraea* L.) in Germany. Outbreaks of moth populations in oak forests in Germany have been recorded since the 1880s, with population density oscillations of 7–10 years [13]. Moth's larvae regularly hatch in spring and can severely defoliate oak trees, with visible changes of organic matter fluxes from canopy to soil, but no subsequent tree mortality, presumably due to generation of new shoots on defoliated oak trees. We quantified carbon (C) and nitrogen (N) element fluxes in insect faeces, leaf litter and throughfall during three subsequent years and analysed soil solutions and soil humus layers in uninfested compared with infested forests. In a microcosm incubation experiment, we analysed carbon fluxes in soils under controlled conditions by simulating naturally occurring pest outbreaks (for a list of all quantified elements, see the Material and methods section). We hypothesize that (i) forest pests influence the carbon and nitrogen balances in infested forests and (ii) heterotrophic respiration responds to these biogeochemical changes.

## 2. Material and methods

The 3-year study was conducted in two deciduous forest areas in the low mountain range (301–350 m a.s.l.) of southern Lower Saxony (North Germany), with and without outbreak populations of the winter moth and mottled umber. Forests are dominated by sessile oak with a stand age of approximately 120 years. Field sites show similar conditions relating to climatic conditions and soil type, tree species composition, tree age and density. Three infested and three uninfested plots were chosen within these sites with an area size between 500 and 700 m^2^ each. The percentage of foliage loss in infested forests varied between approximately 65% (year 1), approximately 90% (year 2) and approximately 55% (year 3), with establishing pest populations in the year previous to the study years (monitored by the regional forest authority). By contrast, uninfested forest showed virtually no foliage loss, with nutrients coming down as more difficult decomposable litter at the beginning of the dormant season. Mean annual precipitation is 600–800 mm and mean annual temperature is 9°C, with 340–380 mm and 13.5–15°C in the growing season (May–September), respectively (mean values from 1961 to 1990, data from Germany's National Meteorological Service). Soils have developed from a loess layer from solifluction over Triassic limestone, classified as Luvisol (brown soil) [14]. The study plots were arranged in a paired sample comparison of infested versus uninfested forests that were located within close spatial proximity to each other. Across the 3 years, sampling comprised the quantification of C and N element fluxes by throughfall, leaf litter, insect faeces, measurement of element concentration in soil solutions and measurement of element contents in soil humus layers (for a list of all quantified elements, see below). Insect faeces and leaf litter were collected weekly using nylon nets (12–15 m^2^, mesh size 300 µm, 1 per plot) during the growing season (but not in the dormant season) of each of 3 years. Total annual deposition of leaf litter may, therefore, prove to be higher in uninfested compared with infested forests. Throughfall was collected using five throughfall samplers (20 cm diameter) per plot. Soil percolates were collected using three zero tension humus lysimeters per plot placed underneath the humus layer. Soil humus layers were sampled by using three sting cartridges (0.5 l volume) per plot.

For determination of C and N in solid organic matter in insect faeces and leaf litter, samples were weighed, dried at 45°C and analysed for total C and N content with a Leco CHN 1000 Analyser (Leco Enterprise, Moenchengladbach, Germany). Analyses of throughfall and soil solutions included measurements of C and N concentrations in membrane-filtered dissolved solution (less than 0.45 µm); cellulose-acetate filters (Sartorius, Goettingen, Germany) for analysis of dissolved organic carbon (DOC) and dissolved nitrogen (DN), and total organic carbon (TOC) and total nitrogen (TN) was analysed in unfiltered solutions (particle size between 0.45 and 500 µm) by thermal oxidation with the DIMATOC 100 Analyser (Dimatec, Essen, Germany) [15]. Throughfall nutrient fluxes were calculated from measured water volumes and element concentrations [5]. For analysis of total phenols and amino-bound acids, solid matter (debris) as well as insect faeces and leaf litter were collected in the field and immediately frozen in liquid nitrogen tubes. Subsequently, total phenols as well as amino-bound acids were measured using high performance liquid chromatography in fresh material (for total phenols [16], and for protein-bound amino acids [17]). Results were obtained by multiplication of leaf litter and faeces dry mass with mean concentrations of total phenols and protein-bound amino acids, respectively.

In the microcosm incubation experiment, we simulated the impact of insect outbreaks via application of faeces on the element fluxes of a severely defoliated 120-year old oak stand using forest floors with Oh- horizons originating from the uninfested plots. Forest floors were 5–6 cm thick and classified as raw humus (containing 39.0% C and 1.3% N) with a C/N ratio of 30. For the experiment, forest floors were cut out and transferred in soil core boxes to the laboratory. Subsequently, the soil was dried at 25°C for one week. We used soil columns with a diameter of 15.5 cm and a height of 7 cm. Each column was filled with 200 g dried soil to simulate the forest floor. Insect faeces were applied to half of the microcosm to stimulate their impacts on nutrient turnover. In the two weeks pre-incubation phase all soil columns were irrigated with 60 ml of tap water at the beginning of the experiment and irrigated weekly with volumes that were calculated based on the water-holding-capacity-test of the soil according to Carter & Gregorich [18] and adjusted to the weight loss of the microcosm due to evaporation. The incubation experiment was realized in an air-conditioned chamber at 21°C. Microcosm treatments were supplemented with 103 g dry mass of insect faeces derived from larvae of the oak leaf feeding gypsy moth (*Lymantria dispar* L.). This amount of insect faeces corresponded to amounts measured in the field during a mass outbreak in a severely defoliated 120-year old oak forest with 500–800 kg ha^−1^ faeces input into the system in a period of five months [15]. The faeces was available from another experiment and have been shown to only marginally differ from faeces of other oak leaf feeding species. The faeces in our experiment contained 49.5% C and 3.0% N, resulting in a C/N ratio of 17. Soil gas samples (CO_2_) were automatically taken by the continuous flow system and analysed using a gas-phase chromatograph (GC Shimadzu 14 B, Shimadzu, Duisburg, Germany). Concentrations of DOC and dissolved inorganic carbon in soil percolates (0.45 µm membrane-filtered with cellulose-acetate filters, Sartorius, Goettingen, Germany) were determined by thermal oxidation (Dimatoc 100, Dimatec, Essen, Germany). Element fluxes were calculated by multiplying water volumes with element concentrations across the 7-week study [6].

Canopy-to-soil element fluxes were analysed with pooled sub-samples for each year (*n* = 6) by Kruskal–Wallis tests. Element concentrations in soil solutions (*n* = 42) as well as element levels in soil humus layers (*n* = 50) were analysed by signed rank tests, and carbon effluxes in the microcosm experiment (*n* = 35) by χ^2^-tests. The relation of annual N and C canopy-to-soil fluxes (*n* = 6) was tested using Spearman's rank correlation. In the text, arithmetic means ± s.e. are given.

## 3. Results

**Figure 1.**
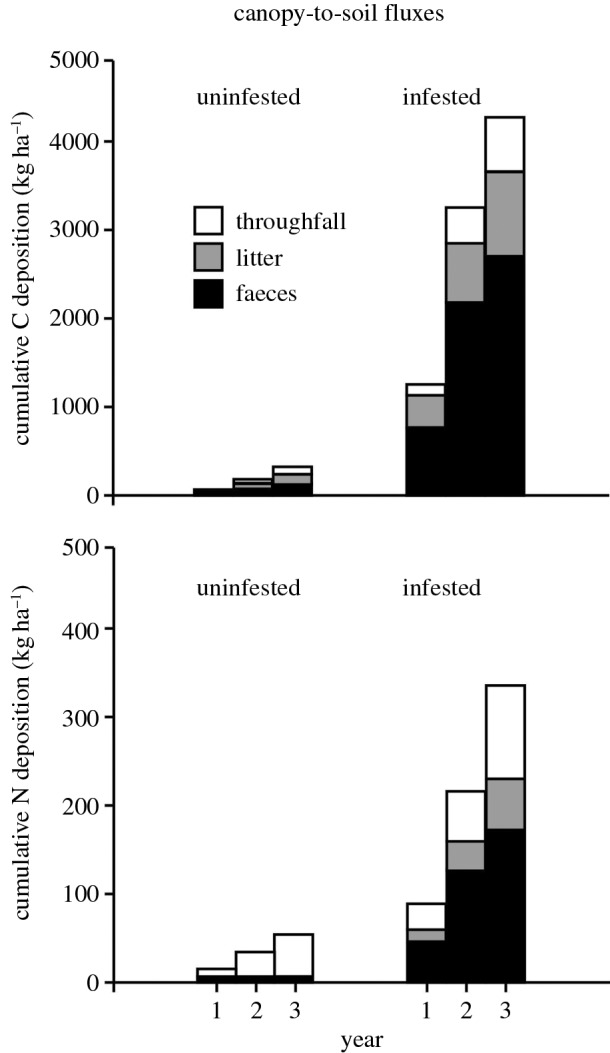
Cumulative fluxes of carbon (C) and nitrogen (N) in uninfested versus infested forests (kg ha^−1 ^yr^−1^) across 3 years.

The deposition of C and N via throughfall, litter and insect faeces varied considerably between uninfested and infested forests; they averaged 142 ± 38 versus 1444 ± 260 kg C ha^−1^ yr^−1^ and 19 ± 2 versus 106 ± 12 kg N ha^−1^ yr^−1^ during the growing season (May–September) across 3 years, respectively. C and N fluxes were significantly higher in infested forests (*p* < 0.05), with faeces contributing most to canopy-to-soil fluxes (figure 1). C and N fluxes were positively correlated (*p* = 0.048, *r_S_* = 0.887, *n* = 6), i.e. annual C deposition increased as annual N deposition increased.

**Figure 2.**
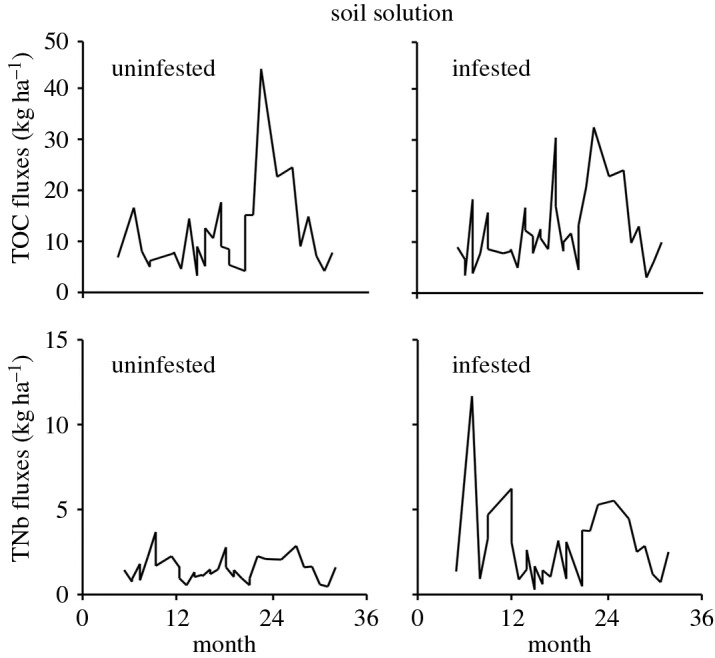
Fluxes of total organic carbon (TOC) and total nitrogen bound (TNb) in soil solutions in uninfested versus infested forests on a monthly basis. Monthly TNb fluxes were significantly higher in infested forests (*p* < 0.001), while monthly TOC fluxes did not differ between uninfested and infested forests (*p* = 0.886).

Fluxes of total TOC and TN in soil solution averaged 11 ± 2 versus 10 ± 1 kg TOC ha^−1^ month^−1^ and 1 ± 0 versus 3 ± 0 kg TN ha^−1^ month^−1^ in uninfested versus infested forests, respectively. TOC and TN fluxes varied largely over time. TOC fluxes did not differ between forests (*p* = 0.885, *n* = 30), while TN fluxes showed consistently higher values in infested forests (*p* < 0.001, *n* = 30; figure 2); 93% of TOC in soil solutions was DOC and 61% of TN was nitrate-N (NO_3_-N).

**Figure 3.**
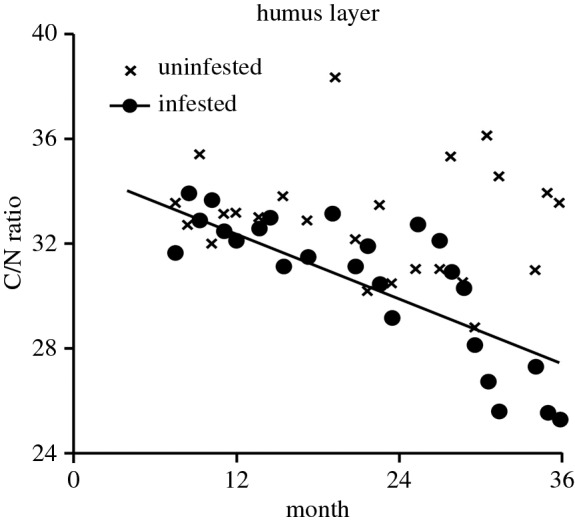
Carbon to nitrogen (C/N) ratio in humus layers of uninfested versus infested forests on a monthly basis. Regression line is shown for descriptive purpose.

C and N levels in the humus layer averaged 38.0 ± 0.5% and 1.3 ± 0.03%, respectively, resulting in a C/N ratio of 31.4 ± 0.4. C levels did not differ between forests sites (*p* = 0.747, *n* = 50). By contrast, in infested forests, N levels were higher compared with uninfested ones (*p* = 0.018, *n* = 50), i.e. they increased over time, thereby significantly scaling C/N ratios down (*p* < 0.004, *n* = 25, figure 3).

In the microcosm incubation experiment, the summarized C effluxes across the 7-week study period were 30.6 ± 0.25 kg CO_2_-C ha^−1^and 21.5 ± 0.30 kg DOC-C ha^−1^ in treatments without added insect faeces (control) and significantly lower than those of treatments with added insect faeces (simulated infestation), with 497.8 ± 14.98 kg CO_2_-C ha^−1^ and 224.8 ± 3.06 kg DOC-C ha^−1^ (table 1). Budgeting these experimental carbon inputs and carbon outputs, the input of C accompanied by N via faeces accelerated a 16-fold output of CO_2_-C and a 10-fold output of DOC-C.

**Table 1.** Fluxes of carbon dioxide carbon (CO_2_-C) and dissolved (DOC-C) in a microcosm incubation experiment simulating an infestation of a defoliating forest pest by adding insect faeces (infested) or not adding insect faeces (uninfested). Measured values were converted to kilogram per hectare (arithmetic means ± s.e.).

	CO_2_ efflux (kg C ha^−1^)		DOC efflux (kg C ha^−1^)	
week	uninfested	infested	uninfested	infested
1	4.6 ± 0.01	48.5 ± 0.01	3.2 ± 0.00	39.0 ± 0.12
2	5.4 ± 0.00	144.3 ± 0.02	3.7 ± 0.03	28.2 ± 0.04
3	3.7 ± 0.00	107.6 ± 0.01	4.0 ± 0.01	37.6 ± 0.12
4	4.5 ± 0.01	45.0 ± 0.01	2.3 ± 0.03	20.1 ± 0.03
5	4.8 ± 0.00	58.0 ± 0.01	2.2 ± 0.02	27.3 ± 0.06
6	4.1 ± 0.00	56.8 ± 0.02	2.4 ± 0.02	43.3 ± 0.04
7	3.5 ± 0.01	36.9 ± 0.01	3.8 ± 0.002	29.3 ± 0.05
total	30.6 ± 0.25	497.8 ± 14.98	21.5 ± 0.30	224.8 ± 3.06

## 4. Discussion

The analyses of element fluxes in the course of a defoliator forest pest epidemic in oak forests showed considerable C and N inputs via throughfall, litter and insect faeces that enter the soil. These inputs increase concentrations and fluxes in soil solutions, scale down C/N ratios, and thereby enhance the decomposition of organic matter. As insect herbivory alters the chemical quality of litter and increases the rate of nutrient cycling (e.g. [19,20]), pest epidemics are likely to provide an important contribution towards accelerated soil respiration in forests where they occur. The high values of DOC and soil CO_2_-C efflux in our experimental microcosms as well as high NO_3_ values in soil solutions with infestation support this finding. A significant decrease in C/N ratio in the humus layers of infested forests was observed after 3 years of infestation, after N deposition rates of greater than 300 kg N ha^−1^. This decomposition rate might indicate a threshold value of N saturation in forests, a state in which the dominance within the soil microbiological community shifts from fungal/mycorrhizal to bacterial, with increased nitrification rates, nitrate leaching and changes in carbon sequestration [21–24].

Few studies suggest a positive feedback between climate change, forest pests and the C cycle. During large-scale outbreaks of xylobiontic insects, extensive tree mortality leads to decreases in C uptake for biomass production and increases in CO_2_ emissions from the decomposition of these trees, thereby altering C cycling for decades [4,25–27]. By contrast, outbreaks of defoliating and sap-sucking insects or pathogens) decrease C uptake for biomass production for only one or a few years, with a recovery of biomass production in subsequent years [24]. In our oak forests, defoliation also did not result in tree mortality, which may be related to the ability of oaks to generate new shoots subsequent to feeding events. Species identity, fluctuations of population densities and their interactions with abiotic and biotic stress factors may distinctly vary forest C cycling in many situations. The increased C and N deposition found during a defoliator outbreak appears to provide large amounts of labile organic C and N, both of which in turn positively influence decomposition and element leaching.

Many studies have analysed N deposition from anthropogenic sources and their impact on forest C. Overall, N can stimulate tree growth in most (but not all) forests, meaning that more C is stored in tree biomass (e.g. [28,29]). Though autotrophic respiration from the rhizosphere is also stimulated by N deposition and generally may increase soil CO_2_ effluxes, heterotrophic respiration from microorganisms often decreased with a strong negative effect at highly productive sites, resulting in an overall negative effect on soil CO_2_ efflux as a result of N addition [30]. N-induced reduction in soil respiration is related to shifts in C allocation by favouring above-ground biomass production instead of that of the root system, shifts in microbial communities transforming organic matter into recalcitrant fractions, and/or shifts in microbial syntheses of extracellular enzymes from the degradation of recalcitrant organic matter towards energy-rich, labile fractions (e.g. [31–33]). By contrast, our results reveal a positive effect of N deposition on soil CO_2_ efflux that could be attributed to N deposition via faeces, litter and throughfall during the course of a pest outbreak coupled with the deposition of high amounts of labile organic matter with a narrow C/N ratio, as well as a defoliated forest canopy increasing soil temperatures. These factors may have favoured microbial decomposition and soil respiration (as well as $\text{N}{{\text{O}}_{3}}^{-}$ leaching) in our study (cf. [34–38]).

In conclusion, the role of forest pests in the cycling of elements has to be reconsidered. Our results indicate that forests pests can mediate a positive feedback between climate change and the C cycle via considerable transformation of oak tree biomass into rapidly decomposable soil organic matter, thereby contributing to soil CO_2_ effluxes and nitrate leaching. However, more evidence is required from field studies comparing the relative importance of different pest species and their interactions with abiotic and biotic factors at varied environmental conditions at which they occur for validation (e.g. changing temperature and precipitation patterns). For example, wood borers and invasive pests may have a larger negative impact on primary production (and forest resilience) compared with defoliators, with large-scale tree mortality that reduces forest carbon uptake and increases future emissions from the decomposition of dead trees [3,4]. Also, more detailed knowledge is needed on the *in situ* conditions as there are series of local interactions that may generate positive and negative feedback effects on the storage of soil organic C. More generally, soils with high pH values and high oxidative and mineral activities appear to be rather ‘substrate-limited’, with limited accumulation of soil organic matter, while soils with opposing characteristics tend to be ‘microbial activity-limited’ and favour the storage of soil organic matter [33]. Overall, forest pests become an increasingly important factor to be considered in C cycling and C sequestration, as climate change appears to be a major driver of pest epidemics that may mediate considerable C cycle feedbacks.

## Data accessibility

The datasets supporting this article are available from the Dryad Digital Repository at http://dx.doi.org/10.5061/dryad.68h07 [39].
